# Evaluation of PCR procedures for detecting and quantifying *Leishmania donovani* DNA in large numbers of dried human blood samples from a visceral leishmaniasis focus in Northern Ethiopia

**DOI:** 10.1186/1471-2334-13-153

**Published:** 2013-03-27

**Authors:** Ibrahim Abbasi, Samar Aramin, Asrat Hailu, Welelta Shiferaw, Aysheshm Kassahun, Shewaye Belay, Charles Jaffe, Alon Warburg

**Affiliations:** 1 Department of Microbiology and Molecular Genetics, The Institute for Medical Research Israel-Canada, The Kuvin Centre for the Study of Infectious and Tropical Diseases, The Hebrew University - Hadassah Medical School, The Hebrew University of Jerusalem, Jerusalem 91120, Israel; 2 Department of Microbiology, Immunology & Parasitology, Faculty of Medicine, Addis Ababa University, PO Box 9086, Addis Ababa, Ethiopia; 3 Department of Microbiology, Immunology & Parasitology, College of Health Sciences, Mekele University, Mekele, Ethiopia

**Keywords:** Asymptomatic infections, Cohort study, DNA extraction, Ethiopia, Visceral Leishmaniasis, *Leishmania donovani*, kDNA-PCR

## Abstract

**Background:**

Visceral Leishmaniasis (VL) is a disseminated protozoan infection caused by *Leishmania donovani* parasites which affects almost half a million persons annually. Most of these are from the Indian sub-continent, East Africa and Brazil. Our study was designed to elucidate the role of symptomatic and asymptomatic *Leishmania donovani* infected persons in the epidemiology of VL in Northern Ethiopia.

**Methods:**

The efficacy of quantitative real-time kinetoplast DNA/PCR (qRT-kDNA PCR) for detecting *Leishmania donovani* in dried-blood samples was assessed in volunteers living in an endemic focus.

**Results:**

Of 4,757 samples, 680 (14.3%) were found positive for *Leishmania* k-DNA but most of those (69%) had less than 10 parasites/ml of blood. Samples were re-tested using identical protocols and only 59.3% of the samples with 10 parasite/ml or less were qRT-kDNA PCR positive the second time. Furthermore, 10.8% of the PCR negative samples were positive in the second test. Most samples with higher parasitemias remained positive upon re-examination (55/59 =93%). We also compared three different methods for DNA preparation. Phenol-chloroform was more efficient than sodium hydroxide or potassium acetate. DNA sequencing of ITS1 PCR products showed that 20/22 samples were *Leishmania donovani* while two had ITS1 sequences homologous to *Leishmania major*.

**Conclusions:**

Although qRT-kDNA PCR is a highly sensitive test, the dependability of low positives remains questionable. It is crucial to correlate between PCR parasitemia and infectivity to sand flies. While optimal sensitivity is achieved by targeting k-DNA, it is important to validate the causative species of VL by DNA sequencing.

## Background

Visceral leishmaniasis (VL) known as Kala-Azar, is a disseminated protozoan infection caused by eukaryotic intracellular parasites belonging to the *Leishmania donovani* complex. An estimated 390,000 VL cases occur annually, over 90% of which are concentrated in the Indian sub-continent, East Africa and Brazil [1,2]. Distinct modes of transmission characterize the two causative parasite species responsible for VL. *L*. *infantum* in Europe, the Middle East and North Africa and *L*. *donovani. chagasi* in Latin America are transmitted zoonotically with dogs serving as reservoir hosts while *L*. d *donovani* in the Indian subcontinent as well as East Africa is considered anthroponotic and transmitted between humans [3].

In Africa, the worst affected region is southern Sudan with an estimated average of 15,000-20,000 cases per year [4,5]. The most important VL endemic areas in Ethiopia are found in the northwest (Metema-Humera lowland), which accounts for approximately 60% of the cases, and in the southwest (Lake Abaya, Omo river plains and Segen and Woito valleys) [6]. In recent years VL has spread to the highlands of Libo-Kemkem district (south of Gondar), claiming the lives of hundreds of patients [7,8].

Patients with clinical symptoms of VL are routinely diagnosed using either parasitological or serological methods. The former method relies primarily on microscopic examination of stained splenic aspirate smears (96% sensitive). For serological diagnosis of VL and PKDL, two simple tests are used Freeze Dried - Direct Agglutination Test (FD-DAT) and rK39 strip test. A multi-center comparison of these assays demonstrated that while FD-DAT and rK39 tests are highly reliable in the Indian Subcontinent, >95% sensitivity and >90% specificity, they are less useful in Africa [9]. In general, the FD-DAT showed higher sensitivities (86 – 99%) and specificities (82 – 98%) than the rK39 test in Africa (sensitivity 75 – 85% and specificity 70 -92%). However, there was considerable variation in these parameters depending on the origin of the patient (Ethiopia, Kenya or Sudan). The specificity and sensitivity of serological diagnosis can be improved if rK39 and DAT are used in series [10]. However an urgent need exists for better diagnostic tests for VL in East Africa.

PCR-based diagnostic assays are more sensitive than traditional methods including immunoassays [11]. There are several PCR protocols for detecting and diagnosing *Leishmania* infections in humans. These include; kinetoplast DNA (kDNA) minicircles [12,13], the small subunit rRNA gene [14] internal transcribed spacer 1 (ITS1) [15] and spliced leader sequence [14,16]. These PCR systems are genus-specific but do not separate the different *Leishmania* species. Further analysis of the PCR amplicon is required for species identification. For example, restriction cut analysis following PCR amplification of the ITS1 [15,17], high resolution melt analysis of the kDNA / PCR amplicon or the 7SL gene [12,18,19].

As part of a study aimed at elucidating the role of symptomatic and asymptomatic *L*. *donovani* infected persons in the epidemiology of Kala Azar, we are conducting a thorough study of persons living in the endemic district of Tahtay Adiabo in Northern Ethiopia. An important component of the project is the identification of putative parasite reservoirs in VL and PKDL patients as well as asymptomatic (sub-clinical) carriers. Some 4,900 individuals living in 18 villages were screened for infection or exposure to *L*. *donovani* by physical and laboratory tests; Leishmanin Skin Test (LST), Direct Agglutination Test (DAT) and kDNA / RT-PCR. Of the 4,757 dried-blood samples tested by RT-PCR, 680 samples (14.3%) were found positive for *Leishmania* k-DNA (Hailu et al. in preparation). The experiments reported here were performed in order to validate the meaningfulness of the RT-PCR results as indicators for infection with *L*. *donovani*.

## Methods

### Ethical considerations

Informed consent was obtained from all the adults who participated in the study. Consent for inclusion of young children, was obtained from parents or guardians. The study was reviewed and approved by the ethical review committee at the Medical Faculty, Addis Ababa University and the National Research Ethics Review Committee (NRERC) at the Ethiopian Ministry of Science and Technology.

### Samples

As part of a prospective cohort study on the transmission dynamics of VL, blood samples were collected from around 4,900 villagers in the Tahtay Adiabo district of northern Ethiopia. Whole families were selected randomly based on a census comprising over 11,000 individuals.

Four drops (approximately 50 μl each) of venous blood were spotted on Whatman 3MM filter paper. All blood samples were identified by an ID number and processed blindly. To minimize the possibility of contaminating parasite DNA in these PCR procedures all DNA extractions were performed in a room into which, live cultured *Leishmania* were never introduced. The paper punches were washed and sterilized using bleach between different samples. Every RT-PCR run included a negative control (no DNA) and several positive controls with known numbers of parasites (for the standard curve). Only disposable plastic ware (tubes, and pipette tips) was used in all these procedures.

For VL screening DNA was extracted from two paper punch disks (r = 3 mm, calculated to have been saturated with approximately 10 μl of blood each), using a phenol-based DNA extraction method [20]. The results reported in the current publication were derived from re-testing of the original samples (Hailu et al., in preparation).

### Quantitative real-time kinetoplast DNA PCR (qRT-kDNA PCR)

Real-Time hot-start PCR was performed with Absolute Blue qPCR kit (Thermo scientific, Surrey, UK) based on SYBR green detection using a real time PCR thermo cycler (Rotor-Gene 6000, Qiagene, Hilden, Germany). The qPCR reaction (total volume of 20 μl) was prepared by mixing 10 μl of the 2x concentrated absolute blue solution with 1 μM of each kDNA minicircle specific primers JW11 and JW12 (Table 1) and template DNA (2 μl) [12]. For fluorescence signal acquisition, time and temperature profile were set as follow: holding step at 95°C for 15 minutes for enzyme activation, 40 cycles starting in denaturation step at 95°C for 10 seconds, annealing at 58°C for 10 seconds and lastly extension step at 72°C for another 10 seconds. The qPCR kDNA results were viewed and analyzed by the Rotor-Gene’s real time software (Rotor-Gene 6000; Corbett Life Science, Sydney).

**Table 1:** PCR systems and primer sets used for the real time kDNA and ITS1 PCR amplification

**PCR system**	**Primers**	**DNA sequence**	**Amplicon size (bp)**	**Ref.**
kDNA minicircle	JW11	CCTATTTTACACCAACCCCCAGT	120	[12]
	JW12	GGGTAGGGGCGTTCTGCGAAA		
ITS1 PCR	L5.8S	TGATACCACTTATCGCACTT	320	[15]
	LITSR	CTGGATCATTT-TCCGATG		

**Figure 1:**
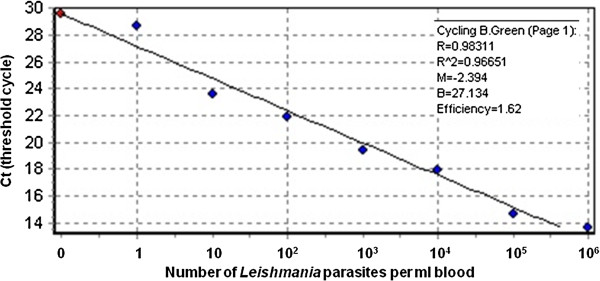
**A standard curve for qRT-kDNA PCR of *Leishmania donovani *promastigotes in blood. **Human blood was mixed well, and dripped onto Whatman 3MM filter papers. On average, each drop (~50 μl) covered an area equivalent to 5 paper punch discs (r = 3 mm). Two discs were used for extracting DNA per reaction (~20 μl of blood). Standard curves were run with every batch of qRT-kDNA PCR and the number of parasites in tested samples was extrapolated from it.

To achieve accurate quantitation, *L*. *donovani* cultured promastigotes were diluted into heparinized human blood at 10^6^, 10^5^, 10^4^, 10^3^, 10^2^, 10, 0 parasites /ml. These parasite dilutions were spotted on Whatman 3MM filter paper and allowed to dry. For every RT-PCR run, two control discs from each concentration were included and the results used to form calibration curves (Figure 1).

### ITS1 polymerase chain reaction (PCR)

PCR reactions were carried out in a volume of 25 μl using ready mix PCR tubes (Syntezza, Jerusalem, Israel). For each reaction 20 pmoles of the two *Leishmania* specific ITS-1 primers (LITSR and L5.8S, Table 1) were added followed by 5 μl of the template DNA [15]. The thermal profile comprised 5 min at 95°C, followed by 35 cycles starting at 95°C for 30 seconds, 56°C for 30 seconds, and 72°C for 1 min, a final elongation step at 72°C for 10 min. PCR results were analyzed by running 10 μl of the PCR amplicon on 1.2% agarose gels with known controls.

### DNA preparation

Fifty nine blood samples that were found positive for *Leishmania* in the cohort study were divided into three categories: 16 samples with 11–100 parasites/ml (low), 24 samples with 100–1000 parasites/ml (medium) and 19 samples with over 1000 parasites /ml (high). Two discs with dry blood (6 mm diameter, Whatman 3MM blotting paper) were cut from each sample with a standard paper- punch. DNA from these discs was prepared using three methods: 1) Phenol/chloroform DNA extraction (repetition of the approach used in the initial study). 2) NaOH based DNA extraction. 3) Potassium acetate DNA extraction method. Precipitated DNA from all samples was suspended in 100 μl of DNAase/RNAase free double distilled water.

#### Phenol based DNA extraction method

The blood/paper discs were incubated in a microfuge tube with 200 μl of lysis buffer (50 mM NaCl, 10 mM EDTA, 50 mM Tris–HCl pH 7.4, 1% triton X-100, and 200 μg/ml of proteinase K) at 60°C for 2 hours. Equal volumes of TE-saturated phenol (pH 8) were added to the aqueous solution, the mixture was vortexed for few seconds and then centrifuged for 2 minutes at 14,000 rpm. The upper aqueous layer was removed to a new micro centrifuge tube and the DNA was precipitated by adding NaCl to a concentration of 0.2 M (addition of 8 μl of 5 M NaCl to 200 μl aqueous solution) and 2.5 volumes of 100% cold ethanol. DNA was incubated at -20°C overnight and centrifuged at 14,000 rpm for 10 minutes. The supernatant was discarded and the DNA pellet was dried in speed-vac.

#### Sodium hydroxide DNA extraction method

The blood/paper discs were incubated in a microfuge tube with 200 μl of lysis buffer (1 N NaOH, 0.1% SDS) at 60°C for 2 hour. The solution was neutralized with concentrated (36%) HCl solution by adding about 18 μl to reach pH of 5–7 as measured using pH-detection strips. Removal of denatured debris was achieved by centrifugation for 10 minutes at high speed in a micro centrifuge. The DNA was further purified by ethanol precipitation as described above

#### Potassium acetate DNA extraction method

Was performed as described by [21]. The blood/paper discs were incubated in a microfuge tube with 200 μl of lysis buffer (1% sodium dodecyl sulfate, 25 mM NaCl, 25 mM EDTA), and samples were placed at 65°C for 2 hours. 100 μl of 3 M potassium acetate (pH 7.2) were added, the mixture was incubated on ice for 30 min and centrifuged at high speed for 15 min in a micro centrifuge. DNA from the supernatant was precipitated by the addition of 600 μl of 100% ethanol.

## Results

### Re-examination of blood samples from the cohort study

The qRT-kDNA PCR results of the cohort study indicated that 69% of the positive samples had 1–10 parasites /ml of blood. These comprised almost 10% of the volunteers. Notably too, the qRT-kDNA PCR values corresponding to parasite concentrations of 10^6^-10^2^ *L*.*donovani* pros/ml of blood in the calibration curves, fit squarely on the linear logarithmic curve, while the lower concentrations below 10 pros/ml deviated significantly (Figure 1). Results were interpreted as showing that low concentrations were less consistent and, therefore not as robust as the higher parasite concentrations. Based on these observations we decided to re-examine some of the samples using the same methodology as that used during the cohort study, namely phenol-based DNA extraction and qRT-kDNA PCR to assess for the possibility of false positives. Results show that 96% to 100% of the samples with high infections (100–1000 and over 1000 parasites /ml, respectively) remained positive on retesting. However, only 85.4% of the samples with 11–100 parasites /ml and 59.3% of the samples with 1–10 parasites /ml were positive again during repeat examination. In addition, 8.4% of the previously negative samples tested as low positives upon repeat PCRs (Table 2).

**Table 2:** Reexamination of qRT-kDNA PCR results from the cohort study

**1**	**2**	**3**	**4**	**5**
**Category parasites /ml**	**1st cohort study kDNA RT-PCR**	**Retested by kDNA RT-PCR**	**kDNA RT-PCR + (current study)**	**Level of uniformity**
0	4,076	107	9	91.6%
1-10	468	108	64	59.3%
11-100	93	48	41	85.4%
101-1000	96	24	23	95.8%
Above 1000	23	19	19	100%

Some of the qRT-kDNA PCR results from the cohort study (Column 2) were reexamined using the same t2:2 protocols (Column 3) the samples that were positive upon re-examination depicted in Column 4. Levels of uniformity (Column 5) indicate the percentage of samples that gave the same result in both tests. Negative and high positive samples were highly consistent. Very low positive samples (1–10 parasites/ml) less so.

### Efficiency of DNA extraction

**Figure 2:**
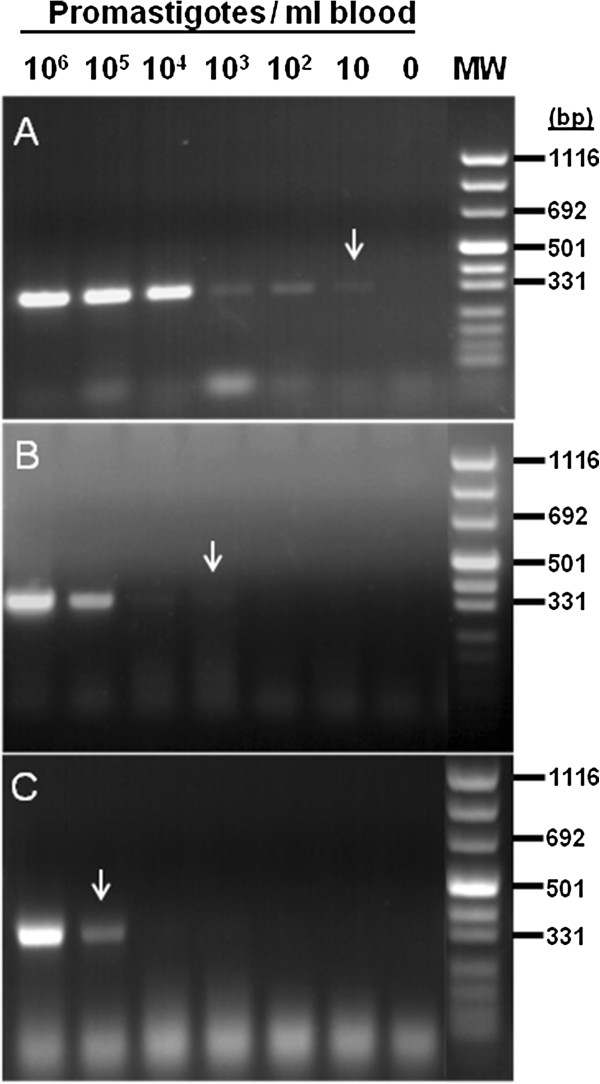
**Comparison of ITS1/PCR results using template DNA prepared by: A) The phenol-based method. B**) The sodium hydroxide-based method. **C**) The potassium acetate method. DNA was prepared from two Whatman 3MM filter paper discs with dried blood containing *Leishmania donovani *promastigotes. 1 ml human blood was spiked with different numbers of promastigotes: lane 1 = 10^6^, lane2 = 10^5^, lane 3 = 10^4^, lane 4 = 10^3^, lane 5 = 10^2^, lane 6 = 10, lane 8 = 0.

In order to determine the efficacy of simple inexpensive DNA preparation protocols for detecting *Leishmania* DNA in dried blood samples, DNA was prepared using phenol, sodium hydroxide or potassium acetate. The purified DNA was used as template for ITS-1 PCR amplification. The phenol-based method yielded the best template, allowing detection of 10 parasites /ml of blood (Figure 2A arrow). DNA prepared using the sodium hydroxide-based method was 3 fold less sensitive requiring a minimum of 10^3^ parasites /ml of blood (Figure 2B arrow). DNA prepared using the potassium acetate-based method proved the least sensitive detecting only 10^5^ parasites /ml of blood (Figure 2C arrow).

In subsequent experiments we re-examined positive blood samples from some of the volunteers from the cohort study. DNA was prepared using the above three methods and tested by ITS1-PCR as well as qRT-kDNA PCR. Tested samples included all infection categories. Here again, DNA preparation using the phenol-based method proved superior and more consistent than the other two techniques (Table 3).

**Table 3:** **Comparison of the efficiency of three DNA preparation methods (phenol, sodium hydroxide and potassium acetate) for detection of *Leishmania *DNA in dried blood spots**

**Infection category**	**Number of samples**	**DNA preparation method**	**kDNA/RT-PCR +**	**ITS1/PCR+**	**kDNA/ITS1**
					**(shared positives)**
Low (1–100)	16	Phenol	13	13	10
		NaOH	12	3	3
		Potassium acetate	3	0	0
Medium (100–1000)	24	Phenol	23	12	11
		NaOH	20	6	5
		Potassium acetate	1	0	0
High (above 1000)	19	Phenol	19	13	13
		NaOH	13	9	8
		Potassium acetate	8	6	6
Totals	59	Phenol	55	38	34
		NaOH	45	18	16
		Potassium acetate	12	6	6

Results for ITS1 were obtained on gels following standard PCR. Data for kDNA was obtained by qRT-PCR.

### DNA sequencing of the ITS1-PCR amplicon

**Figure 3:**
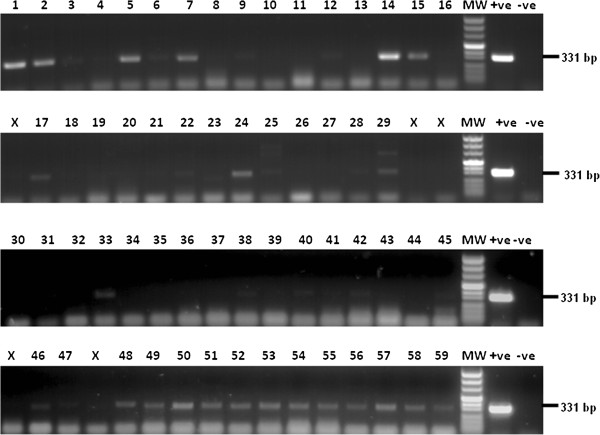
**ITS1 PCR targeting leishmanial DNA extracted from 64 dry blood samples previously shown positive by qRT-kDNA PCR. **DNA from two 6 mm punch discs per specimen, was prepared by the phenol-based method. Twenty-two samples were clearly positive and these were sequenced to determine the *Leishmania *species. Note: low molecular weight bands represent primer-dimers.

To validate the identity of the *Leishmania* DNA in the blood of the naturally-infected volunteers in the cohort study, 64 samples with differing parasite loads were amplified by ITS1-PCR (Figure 3). The amplified PCR products from 16 samples exhibiting moderate to strong ITS1 bands were sequenced by-automated fluorescent DNA sequencing using ABI PRISM 377 sequencer (PE Biosystems, Foster City, California). To improve ability to sequence low parasitemias, ITS1 PCR products from a further 6 samples with weaker bands, were cloned into CloneJet PCR cloning kit (Fermentas, Vilnius, Lithuania). DNA from the produced recombinant plasmids was purified using miniprep purification kit (Qiagene, Hamburg, Germany) and sequenced. The sequences were compared for their homology to known sequences in the GenBank data base using BLAST online service provided through the PubMed /US National Institute of health. Of the 22 samples sequenced, 20 revealed complete homology with *L*. *donovani* ITS1, the other two samples were found to be homologous to *L*. *major* (Table 4).

**Table 4:** **ITS-1 sequencing for validation of *Leishmania *species identity in kDNA / RT-PCR positive samples**

**Number**	**Parasite /ml**	**Identified species**	**Notes**
1	56	*L.major*	Sequencing of cloned ITS1 amplicon
2	87	*L. donovani*	
3	65	*L. donovani*	
4	36	*L. donovani*	
5	23	*L. donovani*	
6	1993	*L. donovani*	
7	69	*L. donovani*	Direct sequencing
8	552	*L.major*	
9	577	*L. donovani*	
10	584	*L. donovani*	
11	643	*L. donovani*	
12	1022	*L. donovani*	
13	1180	*L. donovani*	
14	1314	*L. donovani*	
15	1397	*L. donovani*	
16	8923	*L. donovani*	
17	11735	*L. donovani*	
18	11753	*L. donovani*	
19	11973	*L. donovani*	
20	11988	*L. donovani*	
21	30770	*L. donovani*	
22	47851	*L. donovani*	

Twenty two samples with differing parasite loads were selected for amplification and sequencing. 20/22 samples proved to be *L. donovani* while two were *L. major* infections.

## Discussion

Large-scale cohort studies on infectious diseases in rural areas of Africa are labor intensive and time consuming. Therefore, the samples collected are extremely valuable and the data derived from them warrants rigorous validation. An optimal combination of a sensitive PCR assay with an efficient DNA extraction method is crucial for the success of DNA-based epidemiological studies. There is a wide range of available commercial kits for DNA extraction most of which depend on proteoloytic tissue digestion followed by DNA binding and elution through glass membranes. Although these efficiently produce clean DNA, they are prohibitively expensive when large numbers of samples require processing. We tested three simple DNA extraction methods (costing less than 10% of the cost of commercial kit.) and found that phenol based DNA extraction was by far the most satisfactory, consistently producing good quality template for our qRT-kDNA PCR diagnostic assays.

PCR -based methods for detecting parasites are highly sensitive and have the added advantage that they may be performed on dry specimens without the need for cold-storage [22,23]. In order to optimize our accomplishments from the current cohort studies, we experimented with primers for ITS1 and 7SL RNA gene [17,18]. However, the levels of sensitivity were inadequate (data not shown). Therefore, we resorted to kDNA RT- PCR which is the most sensitive method for detecting *Leishmania* since there are 10,000 kDNA minicircles per parasite [19]. In our hands the limit of detection of the qRT-kDNA PCR was around 10 parasites per ml (Figure 1). For DNA extraction we routinely used 2 punch-disks containing approximately 20 μl of blood (0.2 parasites). The DNA solution was diluted into 100 μl of which only 2 μl were used for each kDNA RT-PCR reaction. Thus, the detection threshold was approximately 0.004 parasites per reaction. This sensitivity is comparable with that previously reported for kDNA RT- PCR in dried blood [12,24]. Since PCR amplification of kDNA using the primers JW11 and JW12 does not discern between *Leishmania* species [12], we amplified and sequenced the ITS1 gene of select samples. As expected, most were shown to be *L*. *donovani*. However, two of the 21 ITS1 PCR sequences were homologous with *L*.*major* (Table 4). This result was surprising for several reasons. Firstly, in a preliminary census of more than 11,000 inhabitants of the Sheraro region, we did not record any cutaneous leishmaniasis cases. Secondly, our entomological studies, which have been going on for over 2 years, have identified only very few specimens of *Ph*. *papatasi*, the vector of *L*. *major*. Lastly, *L*. *major* is essentially a skin parasite and is not normally found in the blood. It is important to note that all PCRs were performed in a "clean room" (i.e. containing no possible source of *Leishmania* DNA contamination) and that validation of these findings included repeat extraction of DNA and repeat PCR reactions.

The ITS1 DNA sequences of the different *Leishmania* species are well characterized and available in GenBank. Many authors have submitted these sequences from different parts of the world. There is a significant sequence difference [exceeding 10%] between *L*. *major* and *L*. *donovani*. The ITS sequences obtained from our samples showed complete homology with either *L*. *donovani* (19 samples), or *L*. *major* (2 samples). The amplified ITS1 sequence was 330 bp and for such short sequence the possible introduced amplification errors caused by DNA polymerase are minimal and would not affect the fidelity of species identification.

Having achieved extremely high sensitivity, it became crucial to validate the repeatability of our qRT-kDNA PCR assay. Only 59% of the samples originally found to contain 1–10 parasites were positive in repeat qRT-kDNA PCR tests (Table 2). This lack of consistency is not surprising since these numbers are very close to the detection threshold of the qRT-kDNA PCR (Figure 1). Indeed, when we randomly re-tested negative samples, over 8% showed up as low positives (Table 2). On the other hand, the results of this study confirmed the overall robustness of qRT-kDNA PCR for detecting *Leishmania* infection in dried blood spots. All 19 samples with high numbers of parasites were confirmed positive upon reexamination using the same protocols. Similarly, of the medium infections, 23 of 24 (96%) were consistently positive. Even lower parasitemias of 11–100 parasites/ml of blood were 85.4% repeatable (Table 2).

As seen in Table 2 the probability of inaccuracies increases around the detection threshold. The first type of error would be a false negative (i.e., missing parasites that do exist in the sample). Such errors could arise from the fact that in the first sample there are no parasites, while in the repeated sample there is parasite DNA. A second type of error is false positive where PCR indicates presence of parasite DNA where there is none. Performing several repeat PCRs on a large number of samples would enable the application of statistical tests to estimate the exact rate of both type of errors and to recalculate the infection rates more rigorously. Since we do not have the material to repeat the tests, we necessarily limit our inferences to the current observation - low qRT-kDNA PCR results (1–10 parasites per ml) are less dependable than either negative or high-positive ones (Table 2).

In terms of disease transmission the most relevant question is which of these PCR positive individuals are infectious to sand flies that imbibe 1.0 μl of blood or less [25,26]. Therefore, to be likely of picking up 1 parasite per meal, they would require infections of 1,000 parasites or more per ml of blood. Although we do not know what is the amastigote dose required for infecting sand flies in nature, laboratory infections of *Ph*. *orientalis*, the vector of *L*. *donovani* in Ethiopia and Sudan required some 2 × 10^4^ *L*. *donovani* promastigotes per ml of blood to obtain a high rate of infection (Seblova et al., in press). Thus, it seems likely that only the very high qRT-kDNA PCR positive individuals actually serve as effective reservoirs for infecting sand flies. This would be consistent with xenodiagnostic data on *L*. *d. chagasi* from Brazil indicating that only patent VL cases were infectious to *Lutzomyia longipalpis* sand flies while asymptomatic carriers were not [27].

In India *L*. *donovani* amastigotes were found in the blood of asymptomatic persons living in endemic regions [28]. In Sudan, *L*. *donovani* was demonstrated in the skin, causing a primary leishmanoma [29]. Asymptomatic infections are thought to be common in Ethiopia as well and may serve as parasite reservoirs [3,30]. However, blood parasitemias may be misleading in that *Leishmania* spp are not “true” blood parasites and may potentially be more abundant in the skin and internal organs. Sand flies macerate the skin to obtain blood. Thus, they may pick up parasites not only from the blood they imbibe but also from resident macrophages in the skin itself. In fact, low amounts of *Leishmania* DNA in the blood may indicate heavy infections elsewhere in the body. To gain an improved understanding of the possible significance of such findings, we plan to test skin samples as well as blood from volunteers in future cohort sampling.

## Conclusions

Our results so far indicate that the detection of very low blood parasitemias is not a reliable parameter for determining infections with *L*. *donovani*. Current studies are focused on following PCR positive volunteers over time to detect possible correlations between the levels of blood parasitemias and the probability of a person developing VL. The cumulative data analyzed using sophisticated statistical methods and examined with a dynamical VL model should help to determine the probable pathogenetic course of asymptomatic *L*. *donovani* infections, either becoming sick or recovering with time.

## Competing interests

The authors declared that they have no competing interest.

## Authors’ contributions

Field work in Ethiopia collection of samples – AH, WS, AK, SB. Conceptual development and optimization of the PCR methodology IA, CJ, AH, AW. Performance of the Real time KDNA assays – IA, SA, WS. Analyses and interpretation of the results - IA, CJ, AH, AW. Writing of the manuscript – IA, SA, AH, AW. All authors read and approved the final manuscript.

## Pre-publication history

The pre-publication history for this paper can be accessed here:

http://www.biomedcentral.com/1471-2334/13/153/prepub
